# Outcomes of a Novel Positioning Technique for Ophthalmic Surgery in Patients With Atypical Anatomy

**DOI:** 10.7759/cureus.94140

**Published:** 2025-10-08

**Authors:** Jordan Jensen, Nikki Gill, William A White

**Affiliations:** 1 Ophthalmology, University of Kansas Medical Center, Prairie Village, USA

**Keywords:** cataract surgery, kyphosis, spinal deformities, surgical outcomes, surgical positioning

## Abstract

In cataract extraction surgery, patient positioning and surgeon comfort are necessary for success. The patient’s head must be flat and parallel to the floor to minimize complications and permit the use of a standard ophthalmic microscope. This position is often difficult in patients with spinal abnormalities, such as age-related hyperkyphosis or the exaggeration of the thoracic spine curvature. Multiple methods have been proposed to improve positioning in patients with spinal abnormalities. While these positions benefit the patient, they are often uncomfortable for the surgeon due to a lack of space or increased difficulty during the surgery. We propose a new surgical stool that, when used with the Trendelenburg position, can optimize patient positioning and surgeon comfort without increasing the risk of complications.

A retrospective chart review was conducted of eight patients (15 eyes) with spinal deformities that necessitated the proposed positioning for cataract surgery. The measured outcomes included best-corrected visual acuity (BCVA), intraocular pressure (IOP), central corneal edema, non-centered lens placement, retained native lens material, and cystoid macular edema (CME). Immediate postoperative outcomes, such as cumulative dissipated energy (CDE), capsular membrane rupture, use of Malyugin ring or iris hooks, and placement of wound sutures, were measured as surrogates of surgery difficulty.

Analysis demonstrated that the outcomes of patients with hyperkyphosis who underwent cataract surgery with the novel positioning were non-inferior to the outcomes of standard cataract surgery on non-hyperkyphotic patients. There was also no apparent difference in qualitative outcomes between the two groups. This pilot study shows that our novel positioning method provides a cost-effective alternative for ophthalmic surgery in patients with hyperkyphosis without increased risk of complications.

## Introduction

Cataract surgery is one of the most common procedures in developed countries, and the rate of surgeries has increased in recent years [1]. This increase can be attributed to an aging population, a lower threshold for visual impairment, and advancements in surgical technology. In the United States, the mean age of patients who receive their first cataract surgery is 67 years [2].

For successful cataract surgery, patients must lie flat, and surgeons should be comfortably positioned temporally or cephalad. This is necessary to visualize the red reflex and prevent increased intraocular pressure [3]. Additionally, complications are more likely to occur if the patient or surgeon is uncomfortable during surgery. Patients with atypical anatomy sometimes cannot lie flat, making the typical surgical positioning impossible. This is the case in patients with age-related hyperkyphosis, prompting a need for modifications in surgery.

Age-related hyperkyphosis is the exaggeration of the normal thoracic spine curvature that usually begins around age 40. It is estimated that at least 20%-40% of patients over 60 years old have hyperkyphosis, and it is more prevalent in women [4,5]. This abnormal curvature causes preferential flexion in the shoulders and hips, leading to an inability to lie flat. This is especially true on the standard ophthalmic surgical gurney, which has led to various proposed modifications.

One such modification is placing the surgical bed in a steep Trendelenburg position [6-9]. This modification has been reported in several cases where patients recovered with good visual acuity and few complications. However, there has not been a prospective analysis of the efficacy and safety of this position. Additionally, observations have been reported of surgeon discomfort due to the small space under the head of the bed for the surgeon’s legs and foot pedals, and the unfamiliar angles. As mentioned above, surgeon comfort is just as crucial in ensuring a successful surgery.

To compensate for this discomfort, others have proposed using face-to-face operations with the patient in a semi-recumbent position or seated in a wheelchair [10-12]. The standard operating microscope can be adjusted for such an angle. These surgeries were successful, and the main complication was cases of displaced lens fragments into the vitreous cavity [13]. There were no reported systemic adverse effects on the patient with this positioning. However, some reports discussed the increased difficulty with this surgery due to the unfamiliar angles and possible discomfort for the surgeon [14].

To combat the issue of little space for the surgeon while the patient is in the Trendelenburg position, we propose the use of a particular surgical stool that is economical and can easily be transferred to other facilities and surgeries (Model LXS-M-HG, Biofit Engineered Products, Bowling Green, OH). The seat places the surgeon in a higher position, allowing optimal distance to the foot pedals without obstruction from the surgical bed.

This article was previously presented as a poster at the Association for Research in Vision and Ophthalmology 2023 conference.

## Materials and methods

**Figure 1:**
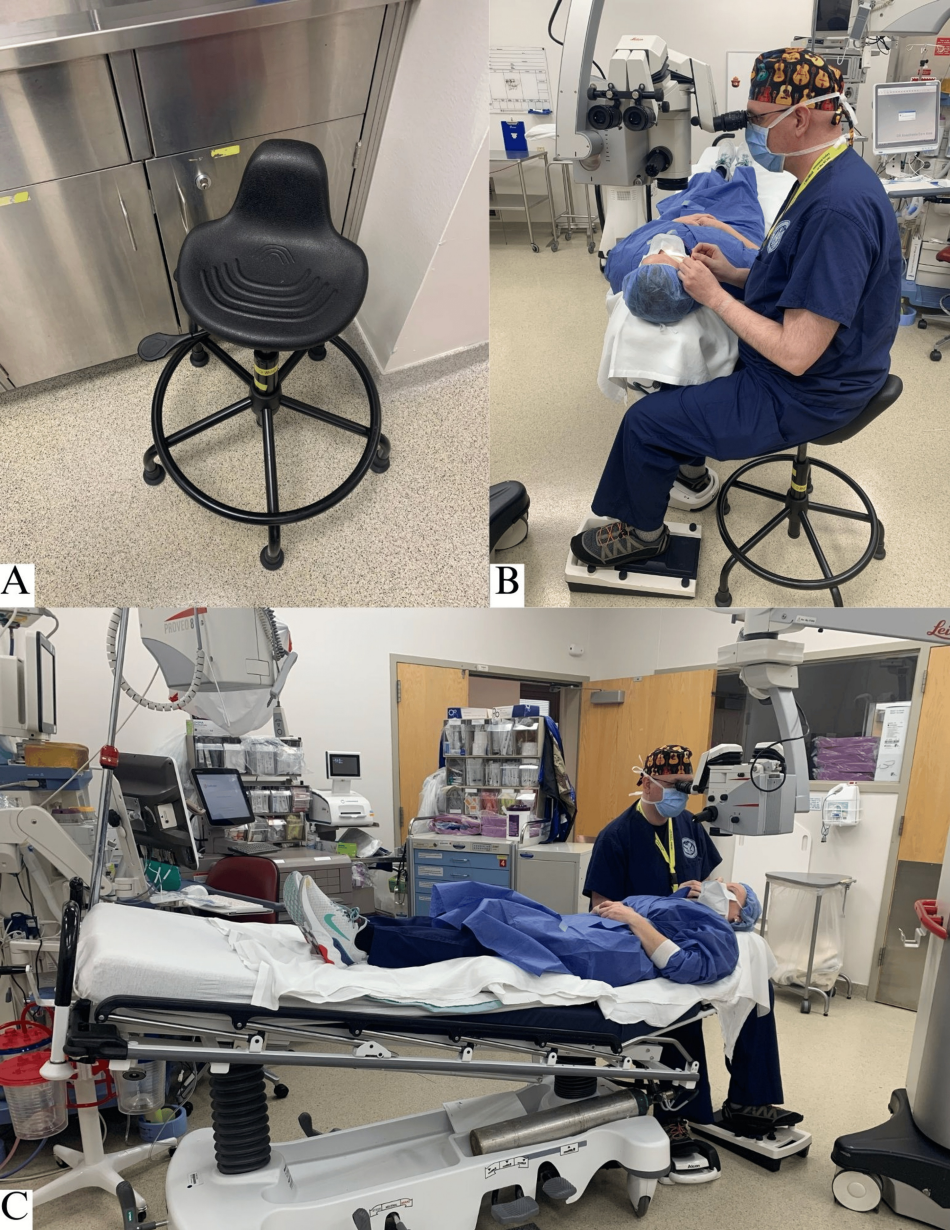
Representative photo of novel positioning with a high-set chair and the Trendelenburg position (A) High-set stool; (B) Representative photo of surgeon positioning; (C) Representative photo of patient positioning.

This study intends to explore a novel method for cataract extraction surgery in patients with spinal deformities. The method specifically combines a high-set stool with a flatter Trendelenburg position compared to studies previously mentioned (Figure 1). We aim to determine whether the efficacy and safety of this method are equal to or non-inferior to the standard surgical setup in patients with normal anatomy. The study was approved by the Kansas City Veterans Affairs Medical Center (VAMC) Institutional Review Board (IRB) with a Health Insurance Portability and Accountability Act (HIPAA) authorization waiver on February 2, 2023 (approval number: 1735614-1). A retrospective chart review was conducted of eight patients (15 eyes) with spinal deformities that necessitated the proposed positioning for cataract surgery. Patients with additional ocular and significant medical comorbidities were excluded from the study to prevent confounding. Such conditions included uncontrolled retinopathy and visually significant glaucoma. These cases were compared to 16 control cases (eight patients, 16 eyes) of standard cataract surgery conducted around the same time frame. The control cases were chosen at random from the same electronic medical record. One surgeon (WAW) performed all cases at a single site (Kansas City VAMC). These surgeries were conducted from October 2019 to November 2022.

Measured outcomes

Patients were followed at postoperative day one (POD1), postoperative week one (POW1), and postoperative week four (POW4). Visual acuity (VA), best-corrected visual acuity (BCVA), and intraocular pressure (IOP) were measured at these visits. BCVA was measured via the Snellen chart, then converted to logMAR, and IOP was measured with handheld tonometry (Tonopen or iCare). Qualitative outcomes measured were the presence of central corneal edema, non-centered lens placement, retained native lens material, and cystoid macular edema (CME). Immediate postoperative outcomes include cumulative dissipated energy (CDE), capsular membrane rupture, use of Malyugin ring or iris hooks, and placement of wound sutures; these outcomes could indicate intraoperative challenges experienced by cataract surgeons.

Statistical analysis

All deidentified patients and outcomes were recorded in a Microsoft Excel sheet (Microsoft Corporation, Redmond, WA). The mean BCVA and IOP of the control and novel groups were calculated for each postoperative visit. The percentage of cases with a presence of each qualitative outcome and immediate postoperative outcome in both groups was also calculated. A two-sample T-test was conducted to determine whether the differences in CDE, BCVA, and IOP were statistically significant, with a p-value significance set as < 0.05.

## Results

Demographics

The majority of patients were male, except for one female. All patients were Caucasian. The ages of patients ranged from 69 to 96 years (median = 75 years, mean = 76 years).

One patient was lost to follow-up for the POW4 visit of the left eye, so those details could not be included in the final POW4 analysis. One patient in the novel group missed their POD1 and POW1 visits due to a fall necessitating hospitalization; this patient was only included in the POW4 analysis. Six eyes in the novel group did not have a record of IOP measurements for POW4, so they were excluded from the POW4 IOP analysis.

Intraoperative outcomes

Table 1 shows the mean intraoperative outcomes, including CDE, capsular membrane rupture incidence, Malyugin ring use, and wound sutures. There were no instances of capsular membrane rupture. The mean CDE was 9.09 in the control group and 11.54 in the novel group. This difference was not statistically significant (t(29) = 1.336, p = .192).

**Table 1:** Intraoperative outcomes CDE: cumulative dissipated energy

Intraoperative measure	Control	Novel Method
CDE mean	9.09	11.54
Capsular membrane rupture	0%	0%
Malyugin ring use	19%	20%
Wound suture placement	6.25%	6.67%

Postoperative BCVA and IOP 

Tables 2, 3 demonstrate the mean and standard deviation of BCVA and IOP at each postoperative visit. The BCVA and IOP between the two groups were not significantly different at any point, with a p-value significance set at p < 0.05. By POW4, all BCVA were logMAR 0.176 or better, apart from one eye in the control group (logMAR 1) and two eyes in the novel group (same patient, both eyes logMAR 0.301). All patients had normal IOPs by POW4. 

**Table 2:** Postoperative BCVA (logMAR) Control and novel values are represented as mean +/- standard deviation. P-value significance set as p < 0.05. POD: postoperative day; POW: postoperative week; BCVA: best-corrected visual acuity

BCVA (logMAR)	Control	Novel Method	t-test (df)	p-value
POD1	0.268 +/- 0.252	0.294 +/- 0.226	t(28) = 0.296	0.770
POW1	0.115 +/- 0.245	0.072 +/- 0.072	t(28) = 0.537	0.596
POW4	0.093 +/- 0.255	0.078 +/- 0.107	t(28) = 0.210	0.835

**Table 3:** Postoperative IOP (mmHg) Control and novel values are represented as mean +/- standard deviation. P-value significance set as p < 0.05. POD: postoperative day; POW: postoperative week; IOP: intraocular pressure

IOP (mmHg)	Control	Novel Method	t-test (df)	p-value
POD1	14.44 +/- 5.70	15.36 +/- 6.09	t(28) = 0.345	0.733
POW1	11.88 +/- 2.63	11.7 +/- 3.93	t(28) = 0.074	0.942
POW4	12.13 +/- 2.16	12 +/- 3.12	t(22) = 0.1	0.921

Qualitative measures

Table 4 shows the measured qualitative outcomes present by the end of the follow-up period (POW4). There were two cases of temporal corneal edema in the novel group, but 0 cases of central corneal edema. There were no instances of intraocular lens (IOL) dislocation, retained lens material, or CME in either group.

**Table 4:** Qualitative outcomes Values represent the percentage of occurrence for each outcome. IOL: intraocular lens; CDE: cumulative dissipated energy

Qualitative measure	Control	Novel Method
Central corneal edema	0%	0%
Well-centered IOL	100%	100%
Retained lens material	0%	0%
CME	0.00%	0.00%

## Discussion

Multiple positioning techniques have been proposed to facilitate cataract surgery in patients with abnormal spine curvatures. Such modifications include a steep Trendelenburg position or face-to-face operations with the patient seated upright [6,7,9-12,15]. However, both positions placed the patient or surgeon at unfamiliar angles, which increases the complexity of surgery and the risk of complications [8,14].

This study found that the addition of a high-set stool to a flatter Trendelenburg position could provide a safe option for cataract extraction in patients with kyphosis. Additionally, this combination does not require additional training of staff in patient preparation. It can at once be deployed and then stored, therefore supporting fast transition between cataract cases. These qualities perhaps represent an advantage over other proposed methods, but a dedicated comparison would be required in future studies.

The results of this study show that our proposed positioning method during cataract extraction provides comparable outcomes to routine surgery in healthy patients. Surgical difficulty, intraoperative complications, and postoperative outcomes were not significantly different between the two groups. Given the ease and accessibility of this positioning method, it can also be studied in ophthalmic surgeries other than cataract extraction.

This study is limited in its small sample size and retrospective nature, so patient comfort could not be studied. Our sample contained only veterans and lacked racial, gender, and ethnic diversity; thus, our results may not be generalizable to the US population. Further analysis could be done to confirm its safety and efficacy on a larger and more diverse population. Additionally, this study does not account for differences in provider comfort and skill, as only one surgeon completed the cases. Future studies should also consider variations in patients with specific comorbidities. For instance, care must be taken in patients with cardiopulmonary disorders or obesity, as these patients are more likely to experience difficulty breathing, increased venous pressure, and increased IOP while in the Trendelenburg position.

## Conclusions

Optimal positioning is a key factor for surgical success, and alternatives must be considered for patients with abnormal spine curvatures. Our proposed positioning method with a high-set stool allows for more space under the table when these patients are in the Trendelenburg position. This pilot study supports that this positioning in patients with spinal abnormalities is equally effective and safe as the standard positioning in healthy patients. Future studies could compare this to other methods for kyphotic patients, in addition to analyzing surgeon comfort. This method should be used with discretion by the primary surgeon, as this study is limited by size and diversity but represents a pragmatic consideration that may result in a successful surgery for an otherwise challenging patient scenario.
